# Characterization of Clinical and Genetic Risk Factors Associated with Dyslipidemia after Kidney Transplantation

**DOI:** 10.1155/2015/179434

**Published:** 2015-04-06

**Authors:** Kazuyuki Numakura, Hideaki Kagaya, Ryohei Yamamoto, Naoki Komine, Mitsuru Saito, Tsuruta Hiroshi, Susumu Akihama, Takamitsu Inoue, Shintaro Narita, Norihiko Tsuchiya, Tomonori Habuchi, Takenori Niioka, Masatomo Miura, Shigeru Satoh

**Affiliations:** ^1^Department of Urology, Akita University Graduate School of Medicine, Akita 010-8543, Japan; ^2^Department of Pharmacy, Akita University Hospital, Akita 010-8543, Japan; ^3^Center for Kidney Disease and Transplantation, Akita University Hospital, Akita 010-8543, Japan

## Abstract

We determined the prevalence of dyslipidemia in a Japanese cohort of renal allograft recipients and investigated clinical and genetic characteristics associated with having the disease. In total, 126 patients that received renal allograft transplants between February 2002 and August 2011 were studied, of which 44 recipients (34.9%) were diagnosed with dyslipidemia at 1 year after transplantation. Three clinical factors were associated with a risk of having dyslipidemia: a higher prevalence of disease observed among female than male patients (*P* = 0.021) and treatment with high mycophenolate mofetil (*P* = 0.012) and prednisolone (*P* = 0.023) doses per body weight at 28 days after transplantation. The genetic association between dyslipidemia and 60 previously described genetic polymorphisms in 38 putative disease-associated genes was analyzed. The frequency of dyslipidemia was significantly higher in patients with the glucocorticoid receptor (*NR3C1*) *Bcl1* G allele than in those with the CC genotype (*P* = 0.001). A multivariate analysis revealed that the *NR3C1 Bcl1* G allele was a significant risk factor for the prevalence of dyslipidemia (odds ratio = 4.6; 95% confidence interval = 1.8–12.2). These findings may aid in predicting a patient's risk of developing dyslipidemia.

## 1. Introduction

Dyslipidemia is a disorder of lipid metabolism and is characterized by elevated levels of lipid in the bloodstream. It is frequently observed in renal transplant recipients [1] and is a risk factor for cardiovascular disease and graft loss [2]. Prior to current immunosuppressive practices, the prevalence of dyslipidemia in renal transplant recipients was over 80% [3]; however, despite new interventions, the risk of disease remains at 44% to 60% [4]. There are a number of previously described risk factors for the development of dyslipidemia in renal transplant recipients, including diet and age; however, a number of therapeutic interventions associated with the transplantation, including steroid, cyclosporine (CyA), and mTOR inhibitor treatments, increase the risk of developing dyslipidemia [5].

Steroids influence cholesterol metabolism by activating acetyl-CoA carboxylase, hydroxymethylglutaryl-CoA reductase, and free fatty acid synthase, decreasing the activity of the low-density lipoprotein (LDL) receptor, and inhibiting lipoprotein lipase [6]. Consequently, steroid-based treatments such as steroid pulse therapy result in an increase of very low-density lipoproteins (VLDL), total cholesterol (TC), and triglyceride (TG) and a decrease of high-density lipoprotein (HDL). To avoid this, an early reduction in steroid treatment has been shown to reduce the incidence of dyslipidemia [7]. CyA is administered to transplant recipients to minimize graft rejection by inhibiting the growth and action of T cells. It has also been shown to inhibit 26-hydroxylase, a key enzyme involved in bile acid synthesis, which results in a decrease of cholesterol secretion into the intestine and a development of dyslipidemia [8]. Tacrolimus (TAC), a calcineurin inhibitor, reduces the incidence of dyslipidemia more than CyA (CyA, 36% versus TAC, 26%) [9]. Treatment with steroids and CyA has a synergistic effect that results in an increase in TC levels [10]. mTOR inhibitors, which are also commonly administered as an immunosuppressant, increase both TC and TG levels and inhibit the metabolism of apolipoprotein B100 in a dose-dependent manner. Inhibition of mTOR leads to a reduction in insulin secretion and insulin-like growth factor, which results in an increase in lipid synthesis within liver cells [11].

Since 1998, renal transplant recipients at our institute, who were all Japanese, have been treated under the same TAC-based immunosuppressive regimen. As is well known, there is marked individual diversity of blood TAC, mycophenolate mofetil (MMF), and steroid concentration after transplantation [12–14]. These genetic polymorphisms may influence individual variations in pharmacokinetics of immunosuppressive drugs. However, the association between drug pharmacokinetics and its related genetic polymorphisms with the prevalence of dyslipidemia under the TAC-based immunosuppression has not been definitively established.

The aim of this study was to determine the prevalence of dyslipidemia in a Japanese cohort and determine clinical and genetic characteristics associated with disease risk within the first year after transplantation under the TAC, MMF, and steroid therapy. A number of genetic polymorphisms that are associated with lipid metabolism have been previously described [15, 16]. In this study, 60 polymorphisms in 38 genes known to be involved in lipid metabolism were examined to determine their association with the development of dyslipidemia.

## 2. Materials and Methods

### 2.1. Patients and Diagnosis of Dyslipidemia

One hundred and twenty-six adult patients who (i) received a renal allograft under TAC-based immunosuppression at Akita University Hospital between February 2002 and August 2011, (ii) had no serious complications, and (iii) maintained graft function for at least one year after transplantation were eligible for this study. From this patient cohort, individuals with high LDL cholesterol levels (≥140 mg/dL), hypertriglyceridemia (≥TG 150 mg/dL), and low HDL cholesterol levels (<40 mg/dL) in the first year after transplantation, or those who required oral statin treatment, were defined as having dyslipidemia.

### 2.2. Immunosuppressive Therapy

Patients initially received combination immunosuppressive therapy consisting of TAC, MMF, and steroid. An initial oral dose (0.15 mg/kg) of TAC and 1.5–2.0 g/day of MMF were administered in two equally divided doses every 12 h at a designated time (09:00 and 21:00). The daily TAC dose was adjusted to achieve a whole blood trough level as previously reported [17]. Methylprednisolone was given concomitantly; a dose of 500 mg on the day of surgery was initially administered and was subsequently tapered to 40 mg/day during the first week, 20 mg/day of prednisolone in the second week, 15 mg/day of prednisolone in the third week, and 10 mg/day thereafter. In the maintenance stage, the dose of prednisolone ranged from 2.5 to 10.0 mg/day based on the immunosuppressive state of each patient. After July 2004, all patients received basiliximab (20 mg) intravenously on the day of surgery and on postoperative day 4. Patients that were ABO-incompatible or were receiving a second transplantation initially received MMF starting in 21 days and TAC and steroids starting 7 days prior to surgery. These patients either underwent a splenectomy at the time of transplantation or were administered rituximab (200 mg/body weight) intravenously (since 2005).

### 2.3. Evaluation of Renal Function

Renal function was evaluated based on the estimated glomerular filtration rate (eGFR). The eGFR was calculated using the following equation established for the Japanese population:

(1) eGFR(mL/min⁡/1.732 m2)  =194×Serum  creatinine−1.094   ×Age−0.287×0.739(if  female).

### 2.4. Evaluation of the Pharmacokinetic Profiles of TAC, MMF, and Steroid

On day 28 after renal transplantation, the whole blood samples were collected immediately prior to and 1, 2, 3, 6, 9, and 12 h after the morning oral administration of TAC, MMF, and steroid. The concentration of TAC in the blood samples was measured by a microparticle enzyme immunoassay (IMx Abbott Laboratories, Abott Park, IL) performed in duplicate, and the concentration of mycophenolic acid, which is active metabolite of MMF, was measured by high-performance liquid chromatography. Pharmacokinetic analysis of prednisolone was carried out with a standard noncompartmental method using WinNonlin (Pharsight Co., Mountain View, CA, USA, version 4.0.1). The pharmacokinetics was estimated as previously reported [12, 17].

### 2.5. Genotyping of Genomic Polymorphisms

DNA was extracted from a peripheral blood sample using QIAamp Blood kit (Qiagen, Hilden, Germany) and was stored at −4°C until analysis. Primer sequences and polymerase chain reaction (PCR) conditions for the analysis of each polymorphism were performed according to previous reports [12, 18–29]. All polymorphisms were analyzed by the PCR-restriction fragment length polymorphism (RFLP) method.

### 2.6. Statistical Analysis

Chi-square tests were used to test categorical data, whereas Mann-Whitney *U* tests were employed to analyze continuous values between groups. *P* values less than 0.05 were considered to be statistically significant. A logistic regression analysis was used to perform multivariate analyses. Clinical and genetic variables were included in the multivariate analyses if their univariate *P* value was less than 0.10. The analysis was performed using SPSS version 19.0 statistical software (SPSS Japan Inc., Tokyo, Japan). To test the population homogeneity of the subjects, the genotype frequencies of each polymorphism were tested against the Hardy-Weinberg equilibrium using a chi-square test.

## 3. Results

### 3.1. Patient Characteristics and Clinical Factors

The mean age of the transplant recipients at the time of transplantation was 47.3 years (range, 21–70 years), the dialysis duration was 48.6 months with a range of 0–420 months, and the mean posttransplant follow-up period was 47.5 months (range, 10–125 months). The primary renal diseases among the patient cohort were chronic glomerulonephritis, including IgA nephropathy (*n* = 50), diabetes nephropathy (*n* = 15), polycystic kidney disease (*n* = 8), pregnancy toxicosis (*n* = 5), reflux nephropathy (*n* = 5), nephrosclerosis (*n* = 3), lupus nephritis (*n* = 3), and nephrosclerosis (*n* = 2). Four cases were defined as “other,” and 31 cases were uncharacterized.

Within the first year after transplantation, 44 patients (34.9%) were diagnosed with dyslipidemia. The clinical characteristics of patients with and without dyslipidemia are summarized in Table 1. Females had a significantly higher prevalence of dyslipidemia over male patients. There were no significant differences in other clinical factors, including the mean body mass index, dialysis duration prior to transplantation, and eGFR at 1 year after transplantation.

### 3.2. Pharmacokinetics of TAC, MMF, and Steroid

An analysis of the pharmacokinetics of the immunosuppressive drugs revealed that the dose per body weight of MMF and steroid was higher in the transplant recipients with dyslipidemia than in patients without the disease at 28 days after transplantation (25.9 ± 7.1 versus 22.3 ± 6.9; *P* = 0.012, 0.20 ± 0.05 versus 0.019 ± 0.03; *P* = 0.023, resp.) (Table 2).

### 3.3. Genetic Associations with Dyslipidemia

The association between having dyslipidemia and 60 polymorphisms in 38 genes was analyzed. These genes included known mediators of lipid metabolism (12 polymorphisms in 9 genes), cytokines (16 polymorphisms in 10 genes), and drug metabolism (32 polymorphisms in 19 genes). In seventeen polymorphisms, there were no variant alleles in this population (Table 3). The genotype frequencies at the glucocorticoid receptor (*NR3C1*)* Bcl1* loci were 78* CC* (61.9%), 45* CG* (35.7%), and 3* GG* (2.4%). Patients with dyslipidemia had a significantly higher frequency of the* NR3C1 Bcl1 G* allele (dyslipidemia, 25, nondyslipidemia, 23) than in those with the* CC* genotype (dyslipidemia, 19, nondyslipidemia, 59) (*P* = 0.001) (Table 3).

### 3.4. Multivariate Analysis

In the univariate analysis, female gender, dose per body weight of steroid and MMF, and* NR3C1 Bcl1 G* allele were associated with dyslipidemia. Multivariate analyses of the genotype data further supported the correlation of the presence of the* NR3C1 Bcl1 G* allele (odds ratio, 4.671, *P* = 0.025) with dyslipidemia (Table 4).

## 4. Discussion

The prevalence of dyslipidemia in renal transplant recipients is highly variable, having been previously reported to range from 44% to 80% [1]. Such variations are thought to be due to differences in the background of the patients or differences in the diagnostic criteria used to define dyslipidemia. Diagnosis of dyslipidemia has traditionally been based on TC, LDL and HDL cholesterol, and TG levels. However, TC levels are not associated with an increased risk of cardiovascular disease in the Japanese population, and therefore, LDL, HDL, and TG levels but not TC values are used as diagnostic criteria in Japan [30]. Given that the criteria may be different among previous reports, careful consideration is needed when results are compared among studies [31].

Improvement in the diagnosis of dyslipidemia will ultimately rely on a unified set of diagnostic criteria to evaluate the presence of the disease. After kidney transplantation, the prevalence of dyslipidemia has been demonstrated to increase over time [5]. However, in somewhat of disagreement, a second study reported that the rate of dyslipidemia decreased after one year after transplantation [32]. These two studies highlight the importance of carefully considering the diagnostic criteria and timeframe in which the evaluation takes place. In this study, patients that presented with LDL cholesterol levels above 140 mg/dL, TC levels above 150 mg/dL, and HDL cholesterol levels below 40 mg/dL in the first year after transplantation, or patients requiring the administration of an oral statin, were diagnosed as having dyslipidemia [30]. Using these criteria, the prevalence of dyslipidemia was found to be 34.9%.

Genetic polymorphisms have been previously investigated in association with renal transplantation, including the development of secondary complications such as diabetes [25] and hyperuricemia [33], as well as their role in the metabolism of immunosuppressive drugs [14]. In multifactorial diseases such as dyslipidemia, genetic polymorphism in genes associated with the disease likely influences the susceptibility towards disease progression. Understanding the association between such polymorphism and the disease could lead to the implementation of genetic tests that predict the risk of developing the disease [25, 33]. In this study, 60 polymorphisms in 38 genes were examined, from which the frequency of the G allele in the* NR3C1 Bcl1* gene was found to be higher in patients with dyslipidemia. The glucocorticoid receptor, which is also known as nuclear receptor subfamily 3, group C, member 1 (NR3C1), is a main regulatory receptor at the hypothalamic-pituitary-adrenal axis. Glucocorticoids regulate the release of glucose from cells in order to supply the body response to face environmental stress. They also induce insulin resistance directly by perturbing insulin signal transduction via glucocorticoid receptor and indirectly by promoting visceral fat deposition and loss of lean mass [34]. In the* NR3C1 Bcl1 G* allele carriers, variation in this receptor was associated with obesity, hypertension, and diabetes [35, 36]. Additionally,* NR3C1 Bcl1 GG* genotype was significantly associated with an increased risk of metabolic syndrome and high BMI in Chinese population [37, 38]. It is possible that increased sensitivity of the glucocorticoid receptor [39] has an influence on the development of dyslipidemia [40]; nevertheless, no difference was observed in the steroid pharmacokinetics. Although a level of significance was achieved, the genetic analysis should be interpreted with caution. When multiple testing parameters are taken into account by applying Bonferroni correction (adjusted *P* value < 0.0008 based on 60 single nucleotide polymorphisms analyzed), none of the polymorphisms reached a level of statistical significance. This is not necessarily surprising, given that the study was performed retrospectively and made use of limited patient data and samples that were available. Future studies with a greater number of patients with predefined disease criteria will be important in determining the association of this polymorphism with dyslipidemia.

Apart from the* NR3C1 Bcl1* G allele, a number of predicted risk factors that were initially identified in the univariate analysis were not significantly associated with dyslipidemia when analyzed by multivariate analysis. It is likely that the gender of the patient would have been a confounding factor of the dose of immunosuppressive drugs administered; as pharmacokinetic monitoring of steroids and MMF is not common practice, a fixed dose was administered to all renal transplant recipients regardless of gender or body weight. Consequently, a larger dose per body weight of steroid and MMF would have been administered to women. Conversely, the fact that the area under the concentration, which was an effective pharmacokinetic marker of steroid and MMF treatment [41], was not found to be a significant factor in the multivariate analysis was quite reasonable.

## 5. Conclusion

The prevalence of dyslipidemia was found to be 34.9% in the Japanese cohort analyzed. The pharmacokinetics profiles of TAC, MMF, and steroid were not associated with the development of dyslipidemia under the same TAC-based immunosuppression treatment. However, the frequency of the* NR3C1 Bcl1* G allele was found to be higher in patients having dyslipidemia and therefore may be a putative genetic marker for predicting risk of the disease. Although the mechanism by which the* NR3C1 Bcl1* G allele might be involved in the prevalence of dyslipidemia is not clear, the analysis of dyslipidemia-related polymorphisms may provide a means to predict patient's risk for having dyslipidemia. Further studies with a larger number of subjects are needed to validate the genetic risk factors associated with the prevalence of dyslipidemia and, in particular, the association of* NR3C1 Bcl1* genotypes.

## Figures and Tables

**Table 1:** Comparison of clinical characteristics between patients with dyslipidemia and without dyslipidemia.

	With dyslipidemia (*n* = 44)	Without dyslipidemia (*n* = 82)	*P*
At transplantation			
Age (yrs)	49.2 ± 10.3	46.3 ± 12.8	0.205
Gender (male : female)	21 : 23	57 : 25	0.021
BMI (kg/m^2^)	22.0 ± 3.5	22.6 ± 4.0	0.421
Dialysis duration (mos)	47.9 ± 54.6	49.0 ± 73.9	0.936
Donor age (yrs)	55.7 ± 12.7	58.4 ± 10.1	0.219
ABO incompatible	16	16	0.162
HCV antibody positive	1	2	0.970
1 yr after transplantation			
CMV infection	11	16	0.700
Acute rejection	17	30	0.951
DM	13	22	0.777
Hypertension	32	62	0.773
Hyperuricemia	18	40	0.354
eGFR	54.1 ± 14.1	51.0 ± 14.7	0.306

Chi-square tests were used to test categorical data, whereas Mann-Whitney *U* tests were employed to analyze continuous values between groups.

*P* values less than 0.05 were considered to be statistically significant.

Values are expressed as mean ± SD.

BMI, body mass index; eGFR, estimated glomerular filtration rate; HCV, hepatitis C virus; CMV, cytomegalovirus; DM, diabetes mellitus.

**Table 2:** Comparison of pharmacokinetic parameters (TAC, MMF, and steroid) between patients with and without dyslipidemia.

Drug	Parameters	After one month		*P*
		With dyslipidemia	Without dyslipidemia	
TAC	Dose/BW (mg/kg/day)	0.17 ± 0.08	0.20 ± 0.10	0.679
	AUC_0–12_ (ng·hr/mL)	170.0 ± 34.6	187.4 ± 46.3	0.457
	*C*_max⁡_ (ng/mL)	18.6 ± 4.7	21.1 ± 6.7	0.166
	Trough	9.2 ± 3.2	9.9 ± 3.3	0.518

MMF	Dose/BW (mg/kg/day)	25.9 ± 7.1	22.3 ± 6.9	0.012
	AUC_0–12_ (ng·hr/mL)	46.0 ± 26.8	46.2 ± 18.7	0.611
	*C*_max⁡_ (ng/mL)	9.3 ± 5.5	9.0 ± 4.9	0.716
	Trough	3.9 ± 2.3	2.8 ± 1.7	0.192

Steroid	Dose/BW (mg/kg/day)	0.20 ± 0.05	0.19 ± 0.03	0.023
	AUC_0–24_ (ng·hr/mL)	1051.1 ± 426.9	1059.1 ± 407.4	0.936
	*C*_max⁡_ (ng/mL)	130.9 ± 45.9	141.3 ± 46.5	0.375
	Trough	0.9 ± 2.6	2.4 ± 8.5	0.549

Mann-Whitney *U* tests were employed to analyze continuous values between groups.

*P* values less than 0.05 were considered to be statistically significant.

Values are expressed as mean ± SD.

TAC, tacrolimus; MMF, mycophenolate mofetil; BW, body weight; AUC, area under the concentration-time curve; *C*_max⁡_, maximal concentration.

**Table 3:** Association of pharmacokinetics, cytokines, and dyslipidemia-related polymorphisms.

Number	Gene	Polymorphisms	Categories	OR	95% CI	*P*
1	IL-2	*T-330G *	*G allele *	0.482	0.157–1.480	0.200
2	IL-2R*β*	*C-627T *	*T allele *	0.591	0.187–1.867	0.545
3	IL-4	*C-590T *	*TT genotype *	0.952	0.316–2.873	0.931
4	IL-10	*A-592C *	*C allele *	0.912	0.299–2.784	0.872
5	IL-12B	*A1188C *	*C allele *	1.156	0.272–4.905	0.861
6	TNF-*α*	*G-238A *	*A allele *	—	—	1.000
		*G-308A *	*A allele *	—	—	1.000
7	INF*γ*	*A874T *	*T allele *	—	—	1.000
8	TGF-*β*1	*Codon 10 T/C *	*CC genotype *	1.225	0.322–4.667	0.964
		*Codon 25 G/C *	*C allele *	—	—	1.000
		*C-509T *	*TT genotype *	0.719	0.203–2.543	0.844
9	CRP	*T-717C *	*C allele *	1.474	0.268–8.092	0.993
		*G1059C *	*C allele *	2.289	0.349–15.010	0.686
		*C1444T *	*T allele *	0.926	0.228–3.765	0.804
		*C1846T *	*TT genotype *	1.067	0.358–3.182	0.908
10	HNF1*α*	*Ileu27Leu *	*Leu/Leu genotype *	0.857	0.318–2.310	0.760
		*Ala98Val *	*Val allele *	—	—	1.000
11	Adiponectin	*T45G *	*G allele *	2.000	0.659–6.066	0.341
		*G276T *	*T allele *	0.889	0.236–3.351	0.869
12	PPAR*α*	*Val227Ala *	*Ala allele *	—	—	1.000
13	PPAR*γ*	*Pro12Ala *	*Ala allele *	2.000	0.115–34.824	0.866
		*C161T *	*T allele *	2.036	0.518–7.995	0.565
14	PPAR*γ* coactivator1	*Gly482Ser *	*Ser/ser genotype *	3.250	0.480–21.998	0.438
		*G394A *	*A allele *	0.381	0.073–1.992	0.449
15	Clock gene	*T3111C *	*C allele *	—	—	0.876
16	ACE	I/D	*Deletion *	1.270	0.381–4.230	0.697
17	ATIIR	*A1166C *	*C allele *	0.454	0.105–1.955	0.464
18	KCNJ11	*E23K *	*A allele *	0.571	0.185–1.765	0.329
19	SUR1	*C-3T *	*TT genotype *	0.286	0.081–1.008	0.946
20	UGT1A1	*Promoter TA-repeat *	^*^ *6 allele *	0.729	0.299–1.777	0.486
21	UGT1A6^*^2	^*^ *1/* ^*^ *2 *	^*^ *2 allele *	0.955	0.255–3.576	0.789
22	UGT1A7	*Codon 208* ^*^ * 1/* ^*^ *3 *	^*^ *3 allele *	1.280	0.228–7.187	0.873
23	UGT1A8^*^2	*codon173 A/G *	*GG genotype *	1.176	0.507–2.732	0.705
24	UGT1A8^*^3	*codon277 C/Y *	*Y allele *	—	—	1.000
	
25	UGT1A9	*C-2152T *	*T allele *	—	—	1.000
		*T-275A *	*A allele *	—	—	1.000
		*C1399T *	*TT genotype *	1.126	0.485–2.617	0.783
26	UGT1A9^*^3	*T98C *	*C allele *	—	—	1.000
27	UGT2B7	*C802T *	*T allele *	0.770	0.330–1.795	0.545
28	CYP2C9^*^2	*C430T *	*T allele *	—	—	1.000
29	CYP2C9^*^3	*A1075C *	*C allele *	2.741	0.715–10.509	0.242
30	CYP2C19	^*^ *1/* ^*^ *2/* ^*^ *3 *	^*^ *2/* ^*^ *2, * ^*^ *2/* ^*^ *3, * ^*^ *3/* ^*^ *3 *	0.697	0.220–2.208	0.739
31	CYP3A4	^*^ *1/* ^*^ *18 *	^*^ *18 allele *	—	—	1.000
		*G20230A *	*A allele *	0.551	0.235–1.294	0.169
32	CYP3A5	*A6985G *	GG genotype	1.815	0.773–4.263	0.169
33	MDR-1	*C1236T *	*TT genotype *	1.067	0.291–3.916	0.814
		*C3435T *	*T allele *	0.868	0.200–3.766	0.850
		*G2677T*·*A *	*T or A allele *	0.688	0.152–3.102	0.922
34	OATP1B1	*A388G *	*GG genotype *	1.143	0.482–2.712	0.762
		*G455A *	*A allele *	—	—	1.000
		*T521C *	*C allele *	0.593	0.226–1.551	0.284
		*G721A *	*A allele *	—	—	1.000
35	OATP1B3	*T334G *	*GG genotype *	1.176	0.507–2.732	0.705
		*G699A *	*AA genotype *	0.714	0.242–2.108	0.542
		*Deletion A *	*Deletion *	1.309	0.310–5.534	0.714
36	OATP2B1	^*^ *1/* ^*^ *3 *	^*^ *3 allele *	0.936	0.402–2.176	0.877
37	**Glucocorticoid receptor**	***Bcl I C/G***	***G allele***	**3.375**	**1.568–7.266**	**0.001**
		*ER22/23EK *	*EK allele *	—	—	1.000
		*N363S *	*S allele *	—	—	1.000
38	CES2	*rs2303218 *	*AA genotype *	2.700	0.697–10.465	0.146
		*rs3890213 *	A allele	3.429	0.791–14.854	0.190

IL, interleukin; TNF, tumor necrosis factor; INF, interferon; TGF, transforming growth factor; CRP, C-reactive protein; HNF, hepatocyte nuclear factor; PPAR, peroxisome proliferator-activated receptor; ACE, angiotensin-converting enzyme; ATIIR, angiotensin type 2 receptor; KCNJ, potassium inwardly rectifying channel, subfamily J; SUR, sulfonylurea receptor; UGT, uridine diphosphate-glucuronosyltransferase; CYP, cytochrome P450; MDR, multidrug resistance; OATP, organic anion transporting polypeptide; CES, carboxylesterase; OR, odds ratio; CI, confidence interval.

**Table 4:** Multivariate analysis of risk factors for dyslipidemia.

Factors	Categories	Univariate		Multivariate		
		OR	*P*	OR	95% CI	*P*
*NR3C1 Bcl I *	*G allele *	4.607	0.001	4.671	1.795–12.156	0.025
MMF/BW	≥24.2	2.902	0.027	2.212	0.771–6.350	0.375
Steroid/BW	≥0.188	2.526	0.034	1.126	0.389–3.256	0.843
Gender	Female	2.494	0.021	1.779	0.633–5.000	0.878

NR3C1, glucocorticoid receptor; BW, body weight; MMF, mycophenolate mofetil; OR, odds ratio; CI, confidence interval.
